# Fecal Microbiota Transplantation as a Tool for Therapeutic Modulation of Non-gastrointestinal Disorders

**DOI:** 10.3389/fmed.2021.665520

**Published:** 2021-09-07

**Authors:** Robert Liptak, Barbora Gromova, Roman Gardlik

**Affiliations:** ^1^Institute of Physiology, Faculty of Medicine, Comenius University, Bratislava, Slovakia; ^2^Emergency Department, University Hospital in Bratislava, Bratislava, Slovakia; ^3^Institute of Molecular Biomedicine, Faculty of Medicine, Comenius University, Bratislava, Slovakia

**Keywords:** intestinal microbiota, metabolic syndrome, liver disease, cardiovascular health, autism spectrum disorder, depression, Parkinson's disease, enteric nervous system

## Abstract

Fecal microbiota transplantation has been primarily investigated as a therapeutic tool for a number of gut disorders. Optimistic results from clinical studies on Clostridium difficile infection, inflammatory bowel disease and irritable bowel syndrome have stimulated the expansion of possible indications in which FMT might represent a game changing approach. Microbial dysbiosis was shown in a number of non-gastrointestinal disorders. Moreover, FMT was proven to be effective in therapy of numerous animal models of disease. However, only a proportion of these disorders have been addressed in clinical studies using FMT. These include obesity, non-alcoholic fatty liver disease, cardiovascular inflammation and neurological disorders such as autism, depression and Parkinson's disease. Results from preclinical and clinical studies also outlined possible molecular mechanisms that contribute to alleviation of the disease. These range from increasing the circulating levels of microbial metabolites (trimethylamine N-oxide, lipopolysaccharide, short chain fatty acids) to stimulation of the enteric nervous system. Several methodological shortcomings are still to be addressed; however, positive results of the clinical studies indicate that further investigation of FMT as a therapeutic tool for non-gastrointestinal disorders can be expected in upcoming years.

## Gut Microbiota and Fecal Microbiota Transplantation

Gut microbiota have gained tremendous scientific attention over the last 15 years. With the advances in biotechnology we have been able to, at least partially, describe the microbial environment and its effects on the host. Virtually all parts of the human body have been studied from the microbial point of view. However, the most studied site of the human body remains the gut.

The most abundant members of gut microbiota are bacteria, followed by viruses, archaea, and microbial eukaryotes. Predominant bacterial phyla in healthy individuals are *Firmicutes, Bacteroidetes, Actinobacteria, and Proteobacteria* (1). Interaction between the gut microbiota and the host is closely associated with maturation of the immune system (2), immune homeostasis (3), modulation of xenobiotics (4), and protection against pathogens (5). Gut microbiota dysbiosis either compositional or functional has been linked to autistic spectrum disorder (6), depression and anxiety (7), cardiovascular health (8), metabolic syndrome (9), development of non-alcoholic fatty liver disease (10), chemotherapy effectiveness modulation (11), and even in sepsis (12).

Fecal microbiota transplantation (FMT) is a method for modulating host microbiome in order to restore gut microbiota dysbiosis toward eubiosis. This method is fairly simple, during the procedure healthy stool from a donor is placed into the gastrointestinal system of the recipient via nasogastric tube, colonoscope, capsule or combination of these methods. The first report of FMT in medical literature comes from 1958 and was used to treat pseudomembranous colitis (13). First randomized trial using FMT was conducted in 2013 and since then it has gained more and more attention as an effective tool for alleviating certain maladies (14).

**Figure 1:**
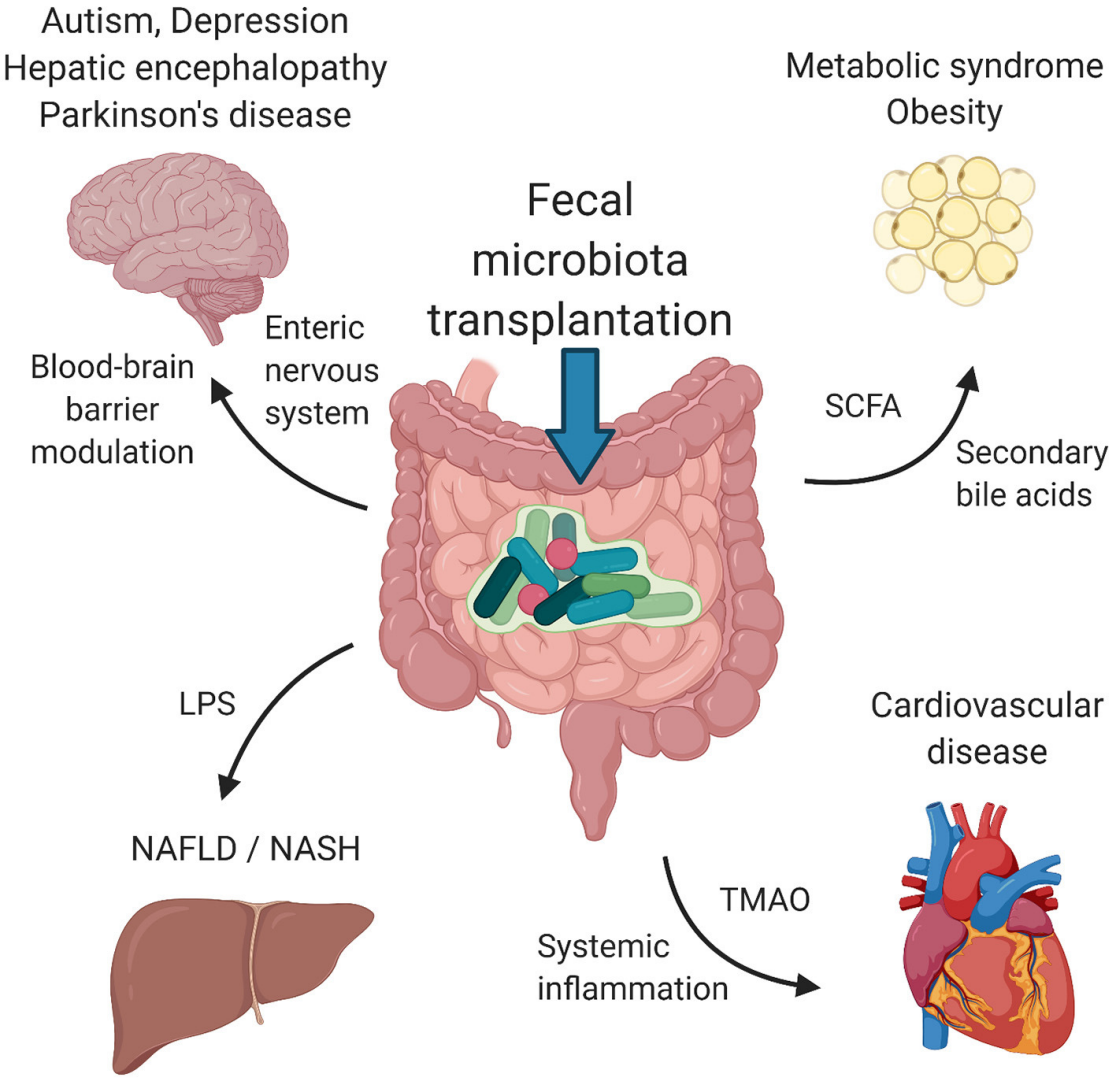
Suggested mechanisms of FMT. Signaling via enteric nervous system and blood-brain barrier modulation by microbial metabolites influence psychiatric/neurological disorders, including autism spectrum disorders, depression, hepatic encephalopathy and Parkinson's disease. Metabolic syndrome and obesity seem to be modulated by the presence of SCFA and secondary bile acids produced by bacteria. LPS and other structural molecules from bacteria entering the portal circulation affect the liver health. Cardiovascular health was found to be regulated by bacterially produced TMAO as well as systemic inflammation induced by the presence of circulating bacteria and their metabolites. FMT, fecal microbiota transplantation; SCFA, short chains fatty acids; LPS, lipopolysaccharide; TMAO, trimethylamine-N-oxide. Created with BioRender.com.

In this review, we summarize the therapeutic applications of FMT for disorders that primarily affect tissues and organs outside the gastrointestinal tract. We focus only on disorders with at least one clinical study using FMT as a therapeutic tool. Studies on metabolic syndrome/obesity, non-alcoholic fatty liver disease/non-alcoholic steatohepatitis, cardiovascular disease, autism spectrum disorders, depression and Parkinson's disease represent a knowledge base for further clinical investigation. Figure 1 depicts the suggested mechanisms by which FMT can modulate the pathogenesis of these disorders. Table 1 summarizes the details of relevant clinical studies employing FMT for the treatment of the reviewed disorders.

**Table 1:** Clinical studies using FMT for non-gastrointestinal disorders.

**Disease**	**Donors**	**Recipients**	**Placebo** **arm**	**Administration** **route**	**Dose of feces**	**Frequency**	**Follow up time**	**Primary outcome**	**References**
Metabolic syndrome	Lean male donors *n = 9*	Obese participants *n = 18*	Yes	Duodenal infusion	500 ml in 0.9% NaCl	One time?	6 weeks	Insulin sensitivity	(15)
Metabolic syndrome	Lean male donors *n = 11*	Obese participants *n = 38*	Yes	Nasoduodenal infusion	500 ml in 0.9% NaCl	One time	6 and 18 weeks	Insulin sensitivity	(16)
Metabolic syndrome	(a) post-Roux-en-Y gastric bypass, *n = 5* (b) metabolic syndrome, *n = 6*	Obese participants (a) *n = 11* (b) *n = 12*	Yes	Duodenal infusion	500 ml	One time	2 weeks	Insulin sensitivity	(17)
Metabolic syndrome	Lean donors *n =* 24	Obese participants *n = 24*	Yes	Oral capsules	15 capsules + 1 capsule weekly	2 days + weekly for 5 weeks	6 weeks	Insulin sensitivity	(18)
Cardiovascular health	Lean vegan donors *n = 10* recipients themselves *n = 10*	Obese participants *n = 20*	Yes	Nasoduodenal infusion	500 ml in 0.9% NaCl	One time	2 weeks	TMAO and PET/CT scan of abdominal aorta	(19)
NAFLD/NASH	Lean donors *n = 21*	Obese participants with hepatic steatosis *n = ?*	Yes	Duodenal infusion	–	Three times at 8-weeks intervals	24 weeks	Liver necrosis score and hepatic gene expression	(20)
Hepatic encephalopathy	Healthy volunteer from OpenBiome	Outpatient cirrhotic men with recurrent HE *n = 20*	No	FMT enema	Frozen-then-thawed FMT units (90 ml total) 2.7 × 10^12^ CFU	One time	5 months	FMT-related serious adverse events (SAEs) and endpoint of death	(21)
Hepatic encephalopathy	Healthy volunteer from OpenBiome	Outpatient cirrhotic men with recurrent HE *n = 20*	No	FMT enema	Frozen-then-thawed FMT units (90 ml total) 2.7 × 10^12^ CFU	One time	15 months from	FMT-related serious adverse events (SAEs) and endpoint of death	(22)
Hepatic encephalopathy	Healthy volunteer from OpenBiome	Outpatient cirrhotic men with recurrent HE *n = 20*	Yes	Oral capsules	15 capsules	–	5 months	Tolerability, FMT-related serious adverse events (SAEs)	(23)
Autism spectrum disorder	Healthy adults	Children with ASD *n = 18*	No	Oral infusion ectal infusion	2.5 × 10^12^ cells/day	2 days – three times per day, 1 h	10–18 weeks	GI and ASD-related symptoms	(24)
Autism spectrum disorder	–	Children with ASD *n = 18*	No	–	–	–	2 years	GI and ASD-related symptoms	(25)
Autism spectrum disorder	Healthy adults	Children with ASD *n = 24*	No	Oral infusion b) rectal infusion	–	–	2 months	GI and ASD-related symptoms	(26)
Depression	Healthy adults	58 years old male 66 years old female 48 years old male	No	Rectal infusion	–	10× over 2 weeks 6× over 1 week 5× over 1 week	6 months 4 years 6 months	GI and depression symptoms	(27)
Parkinson's disease	Healthy adults	PD patients *n = 10* PD patients *n = 5*	No	Rectal infusion nasoduodenal infusion	–	<1 h	1 and 3 months	Motor and non-motor symptoms	(28)
Parkinson's disease	Frozen fecal microbiota was obtained from the China fmtBank	PD patients with constipation, *n = 11*	No	Nasoduodenal infusion	40–50 ml of frozen fecal microbiota in 200 ml of warm normal saline, fresh every time	–	6–12 weeks	16S ribosomal DNA, motor and non-motor symptoms	(29)

## Metabolic Syndrome

Metabolic syndrome as a set of central obesity, dyslipidemia, decreased insulin sensitivity and arterial hypertension has been established in 1988 and has been intensively studied ever since (30). First evidence of gut being at least partly responsible for metabolic syndrome came in 2007 with studies where rodents were fed a high fat diet. After 4 weeks of the diet the rodents showed signs of metabolic syndrome with increased lipopolysaccharide (LPS) concentration in blood. LPS caused a proinflammatory state which decreased insulin sensitivity (31). Landmark studies were performed by Jeffrey Gordon's group, in which they proved that increased adiposity might be a transmissible trait, as was first shown by FMT from *ob/ob* mice into germ free-recipients (32). Association of gut microbiota with obesity was nicely shown in a more recent study where FMT was performed from twins discordant for obesity to germ free mice. Mice that received bacteria from obese twin had increased adiposity and decreased diversity of the gut microbiome (33).

Healthy gut microbiota positively affects host energy metabolism. Bacteria within the gut using their respective metabolic pathways produce molecules that pose a signal for the host cells. Bacteria ferment the indigestible polysaccharides into short chain fatty acids (SCFAs) which act as energy sources for colonocytes but more importantly as signal molecules. SCFAs enhance insulin sensitivity and stimulate fatty acid oxidation and lipolysis (34). Gut bacteria convert primary bile acids into secondary bile acids which affects the farnesoid X receptor, a regulator of host glucose and fat homeostasis (35).

High fructose diet-induced metabolic syndrome in rats was associated with higher abundances of *Coprococcus* and *Ruminococcus* genera. FMT from non-obese healthy rat donors were able to colonize rats fed high fructose diet. Colonization led to reduction of markers of metabolic syndrome and decreased abundance of *Coprococcus* and *Ruminococcus* genera (36). In a similar study diet-induced obese mice received FMT from lean mice. The recipient obese mice were treated with antibiotics prior to FMT to enhance engraftment of donor microbiota. After FMT gut microbiota of obese mice showed greater diversity and regained some functionality showed by metaproteomic approach (37). Similarly, stool from lean mice that exercised transferred into obese mice improved obesity and inflammatory status in obese mice (38). Recent study showed that when autologous stool obtained before induction of obesity is transferred into obese hosts, it results in increased lipolysis and caloric restriction. However, the FMT with caloric restriction group compared to the caloric restriction group without FMT did not show significant difference in gut microbiota composition with only differences *Bifidobacterium* and *Blautia* genera were observed. Authors proposed different mechanisms apart from microbiota engraftment that induced this effect which might include bacteriophages or bacterial metabolites in the stool. However, mice were not observed for long-term effects after the FMT, so the metabolic improvement could have been only temporary (39).

There are several human studies available today. A study by Vrieze et al. showed that FMT from lean donors transferred by single administration via duodenal tube into obese participants increased insulin sensitivity. Obese patients showed decreased gut microbial diversity compared to lean patients. After FMT from lean donors, the gut microbiota diversity was increased significantly. Moreover, sixteen bacterial groups increased in abundance after FMT including potent butyrate producers *Roseburia intestinalis* and *Eubacterium hallii*. Increased butyrate reduces the translocation of endotoxins into the bloodstream, which drives insulin resistance. Whether this is the sole mechanism or there are others at play is currently unknown (15). Subsequently, a similar effect was observed following FMT from lean donors to obese patients via duodenal tube. At 6 weeks after FMT increased insulin sensitivity accompanied by decreased glycated hemoglobin was observed. The gut microbiome changes in patients who responded to FMT showed increased abundance of *Akkermansia muciniphila* and *Eubacterium ventriosum*. There was no difference in gut microbiota diversity among responders and non-responders (16). Another human study had a different design than the previous ones. Stool donors were patients after gastric bypass surgery or obese individuals without intervention. Recipients were obese individuals with metabolic syndrome. The main outcome, insulin sensitivity, showed significant difference, however, this was mainly due to decreased insulin sensitivity of the control group (obese individuals receiving FMT from obese donors). However, a slight increase in insulin sensitivity was observed in the intervention group. The intervention group showed decreased subcutaneous fat inflammation post FMT with decreased expression of chemokine CCL2. The intervention group had increased abundance of *Bacteroides* sp. compared to the control group. In analysis of the intervention group responders and non-responders to FMT were identified. Higher baseline abundances of *Alistipes shahii* and *Anaerostipes hadrus* were associated with better glycemic control after FMT (17).

These three studies, however, are from the same study group. Yu et al. performed double blind placebo controlled pilot trial administering oral capsules of FMT from lean donors to obese individuals. Participants were administered 15 capsules during two consecutive days, followed by a capsule once per week for 5 weeks. The primary outcome was insulin sensitivity measured at week 0 and week 6 and several other secondary outcomes such as HbA1c, body composition, and resting energy expenditure. There were no differences between the intervention and placebo group despite engraftment of donor bacteria as assessed by 16S V4 amplicon sequencing (18). There are several differences between the previous three studies and this one. Most importantly, the route of administration (endoscopy vs. capsule), FMT material (fresh vs. frozen), and colon preparation with laxatives (yes vs. no bowel preparation) was different. Another fact to consider is the geographical region in which these studies were performed (Netherlands vs. USA) which can affect both the donor and recipient microbiota. These questions need to be addressed in future studies.

## Cardiovascular Health

Growing body of evidence has linked gut microbiota to cardiovascular diseases such as atherosclerosis or arterial hypertension (8, 40, 41). Interaction between gut microbiota metabolites and their proinflammatory activity has been suggested. Microbiota metabolism of phosphatidylcholine through the production of proatherogenic metabolite trimethylamine-N-oxide (TMAO). Increased levels of TMAO are associated with increased incidence of major cardiovascular events, as was shown in healthy participants and during a 3 years of follow-up in patients undergoing elective coronary angiography (42). Besides metabolites, gut microbiota dysbiosis with decrease of SCFA producing bacteria may induce systemic inflammation with increased neutrophil infiltration of aortic root, thus exhibiting proatherogenic effect (43). Gut microbiota obtained from donors with hypertension transferred into germ-free mice resulted in increased blood pressure in an animal model (41). Similarly, in high-salt induced hypertension in rats this phenotype was transferable by gut microbiota. Moreover, hypertension was alleviated by transferring healthy gut microbiota. This beneficial effect was accompanied by decreased intestinal derived corticosterone and increased levels of *Bacteriodes fragilis* and arachidonic acid levels in the intestine (44).

Despite this evidence there is relatively small amount of studies exploring the potential effect of FMT to improve cardiovascular health. In a murine model of myocarditis FMT from a healthy donor alleviated myocardial damage by reducing inflammatory infiltration and restoring gut microbiota eubiosis (45). Other authors showed that transplantation of healthy stool to spontaneously hypertensive rats alleviated hypertension via modulation of sympathetic nervous activity (46).

The only human study conducted so far explored the effect of single FMT from vegan donors on TMAO levels and vascular inflammation in a double blind randomized fashion. Recipients received one time only FMT via nasoduodenal tube from lean vegan donors or autologous gut microbiota. After FMT there was no difference in gut microbiota diversity; however, some compositional differences were observed. In the lean donor group, the *Lachnospiraceae* showed increased abundance whereas the autologous group showed increased *Clostridiales* which are known producers of trimethylamine - a TMAO precursor. Vegan donor FMT did not alter fasting or urinary 24 h excretion of TMAO, nor there were changes in vascular inflammation assessed by ^18^F-FDG PET/CT (19).

## Non-Alcoholic Fatty Liver Disease/Non-Alcoholic Steatohepatitis

Non-alcoholic fatty liver disease (NAFLD) is characterized by steatosis affecting at least 5% of the liver volume or weight in non-alcoholic patients. About 30% of people with NAFLD progress into non-alcoholic steatohepatitis (NASH) which is characterized by progressive inflammation. About 20% of patients with NASH will progress into liver fibrosis with decline in liver function (47). The cause of this accumulation is unknown, however, it is often associated with signs of metabolic syndrome (48). The pathophysiology of NASH is poorly understood, however, interaction between genetics, environment, and possibly also gut microbiota is suggested (49). Germ free mice that were fed a high fat diet had a lower rate of liver steatosis than conventional mice, suggesting that gut microbiome might play a role (50). Liver receives the majority of blood supply from the portal vein which drains nutrients along with bacterial compounds from intestines (51). During dysbiosis gut barrier function is disrupted and more bacterial derived compounds enter the circulation, thus the first site these compounds hit is the liver. Afterwards, these molecules, such as LPS, are able to initiate and maintain chronic inflammation. This may potentiate NAFLD and subsequently its progression to NASH (52). Moreover, after transferring gut microbiota from mice with NASH into germ free mice, these mice had more adipose tissue than their counterparts receiving FMT from healthy mice (53).

Gut microbiome changes in NAFLD have been observed, however with conflicting results. Authors found that people with NAFLD and NASH have increased abundances of *Proteobacteria* including increased *Enterobacteriaceae* and decreased *Rikenellaceae* and *Ruminococcaceae* (54). Moreover, some of the bacterial signatures were common with metabolic syndrome and obesity.

In a murine model of diet induced steatohepatitis FMT was successful in restoring gut microbiota dysbiosis. This was accompanied by increased SCFA production and decrease in proinflammatory cytokines production (55).

Recent human double blinded, randomized study investigated the effect of allogenic FMT using stool obtained from individuals eating plant based diet compared with autologous FMT administered three times at 8-weeks intervals via duodenal tube. After the FMT there was no difference in gut microbiota diversity after 24 weeks. However, there were some compositional differences. Individuals receiving allogeneic FMT had increased *Ruminococcus, Eubacterium hallii, Faecalibacterium, and Prevotella copri;* however, the difference did not reach statistical significance. Recipients of allogeneic FMT showed improvement in liver necrosis score which was in line with expression of several hepatic genes including genes responsible for liver endothelial integrity. Changes in gut microbiome might result in decreased levels of microbial aromatic amino acid production, especially phenyllactic acid which is linked to NAFLD. Thus, reducing the production of toxic metabolites by dysbiotic gut microbiota might alleviate NAFLD (20).

## Hepatic Encephalopathy

Under normal physiologic circumstances the gut provides a barrier for various metabolites (e.g., pro-inflammatory molecules, adipokines, TMA etc.) arising in the gut. Metabolites that penetrate this barrier pass through the liver where they are metabolized and thus, the brain is protected from toxic substances. However, in advanced liver disease, such as cirrhosis, these barrier mechanisms are compromised.

Patients with advanced liver disease show gut microbial dysbiosis, increased gut permeability and decreased liver capacity to detoxify toxins. All of which perpetuates one another and ultimately leads to neuronal dysfunction and damage resulting in hepatic encephalopathy (HE) (56).

Patients with HE have reduced abundances of *Lachnospiraceae, Ruminococcaceae* and Clostridiales XIV and increased abundances of *Staphylococcaeae, Enterobacteriaceae*, and *Enterococcaceae* (57). The latter taxa are associated with disease progression and endotoxemia (58). Traditional treatment of HE consisted of lactulose and rifaximin, both of which change bacterial composition without reducing the absolute amount of bacteria in GI tract (59–61).

In a rat model of carbon tetrachloride induced acute liver failure, the rats received FMT with three different concentrations of bacteria or probiotic solution for 3 weeks after acute liver failure induction. All of the rats receiving FMT or probiotics showed increased memory function, improved liver function, decreased intestinal permeability, and reduced ammonia levels and systemic proinflammatory cytokines concentration. However, no analysis of the microbiome was performed (62).

Participants of the first open label clinical trial received a single FMT via enema from a healthy donor. The donor was selected based on relative abundance of *Lachnospiraceae* and *Ruminococcaceae* since these taxa are indicative of gut microbiome health (63). Patients were divided into standard care (SC) group and SC + FMT group. Both groups had 10 participants. SC consisted of lactulose, rifaximin and proton pump inhibitor. FMT patients received antibiotic treatment before FMT. The FMT group had significantly fewer HE episodes and had significant improvement in cognitive function. MELD score was similar in both groups. FMT patients had increased relative abundance of *Lachnospiraceae* and *Ruminococcaceae*. Patients were followed up to 5 months (21). Afterwards, authors decided to expand the follow up period up to 15 months. There were significantly less hospitalizations in the FMT group than SC group and cognitive function was better in the FMT group. Microbiome analysis revealed increased *Burkholderiaceae* and decreased *Acidaminococcus* in FMT patients, however *Lachnospiraceae* and *Ruminococcaceae* were similar between groups (22).

The same authors performed a single center, randomized, single blinded placebo controlled trial with similar design. In this subsequent study FMT was delivered via oral capsules and no pre-FMT antibiotics were administered. FMT patients had fewer serious adverse events, HE episodes, and improved cognitive functions. FMT patients underwent repeated endoscopies which showed decreased expression of IL6, and increased expression of barrier proteins (defensin A5), and E-cadherin in duodenum post FMT. Serum concentration of lipoprotein binding protein also decreased post FMT. Stool microbiota showed increased abundance of *Lachnospiraceae* in the FMT group. Duodenal mucosa in the FMT group showed increase in *Ruminococcaceae* and *Bifidobacteriaceae*, reduction in *Streptococaceae* and *Veillonellaceae* and increased Shannon diversity index post FMT (23).

## Autism Spectrum Disorder

Autism spectrum disorder (ASD) is a neurodevelopmental disorder which results in several behavioral abnormalities. Pathogenesis is unclear, but genes play a major role in developing ASD. However, gene-environment interactions have lately gained more attention in research. Some authors estimate that 50% of the neurobiology is caused by factors that are non-inherited (64). ASD is often accompanied by more or less severe gastrointestinal symptoms. Several studies have shown altered gut microbiota compositions (65, 66). Interestingly, ASD behavior can be transferred via FMT to germ-free mice (67).

In fragile X mental retardation 1 KO mice, a model in which mice elicit autistic like behavior, FMT can ameliorate abnormal behavior in mice (68). In human studies ASD children who received FMT for 8 weeks showed significant behavioral improvement for 8 weeks after the treatment ended (24). In subsequent study by the same author, bowel cleansing, antibiotics, and stomach acid suppressants followed by FMT. Participants were followed by up to 2 years after the treatment stopped. Gastrointestinal symptoms improvement was maintained and behavioral symptoms improved significantly after the treatment ended. Authors observed no adverse effects (25). Although this study was open-label with no placebo control the results are promising. The authors concluded that improvement of gastrointestinal and behavioral symptoms persisted for at least 2 months after FMT compared to the control group. In a conference abstract, a different group of authors showed that FMT in ASD individuals was well-tolerated, improved statistically ASD-related symptoms, and shifted the microbiome of ASD patients toward a healthy state. They reported adverse effects such as fever, allergy, and nausea, but these were mild and transient and could be associated with the mode of delivery of FMT - colonoscopy and gastroscopy. However, there is no information about the pretreatment of recipients and the amount of stool administered (26).

## Depression

Depression has an increasing prevalence in Western world with substantial morbidity and mortality. More and more evidence is emerging associating gut microbiome with depression. The proposed mechanisms include neuroimmune, neuroendocrine and neural pathways (69). For example, mice suffering chronic social defeat stress show depression-like symptoms which are transferable via FMT. *Faecalibacterium rodentium* showed increased abundance in these mice and ingesting this bacterium alone can produce depression-like symptoms. Furthermore, these can be alleviated with subdiaphragmatic vagotomy suggesting that enteric nervous system plays a role (70). Altered gut microbiome composition has been found in patients with depression, a negative correlation between *Faecalibacterium* and depressive symptoms has been found (71). Transferring gut microbiota from depressed humans can induce depression like behavior in rats pretreated with antibiotics (72). Similar result is obtained when transferring gut microbiota from depressed humans into germ free mice (73). Only a small case series described the effect of FMT from a healthy donor into a depressed individual. These patients also suffered from irritable bowel syndrome. FMT was administered via colonoscopy with variable amounts of large bowel enemas based on attending clinician. FMT resulted in alleviating symptoms of both depression and irritable bowel syndrome (27). However, it is questionable whether decreased depression symptoms were the consequence of FMT on depression, or improved symptoms of irritable bowel syndrome.

## Parkinson's Disease

Parkinson's disease (PD) is a neurodegenerative disorder which mainly affects the motor system of the central nervous system. Aggregation of α-synuclein (α-syn) is thought to be the cause of the disease. Dopaminergic neurons in substantia nigra are the first neurons affected by this accumulation. Although multiple gene variants have been associated with the development of PD, the gut microbiome has gained more attention in the last years. The accumulation of α-syn in the enteric nervous system (ENS) has been reported years ago (74). Subsequent study has shown that α-syn is transported via the vagus nerve into the central nervous system after injection into the stomach and duodenal wall (75). *In vivo* studies showed that gut microbiome influences accumulation of α-syn in ENS (76). In mice that overexpress α-syn the presence of gut microbiota is required to promote pathological alterations similar to PD. Moreover, FMT from patients with PD induced PD phenotype in recipient mice (77).

In a murine model of PD FMT was sufficient to ameliorate PD symptoms and increased striatal dopamine and serotonin in recipient mice. FMT also reduced neuroinflammation. In PD mice the authors observed gut microbiota dysbiosis compared to healthy mice. FMT treatment was sufficient to remove these differences and tip the scale toward eubiosis. PD mice showed increased Proteobacteria at phylum level with decreased *Clostridiales* at the order level (78).

A human pilot study including 15 patients receiving FMT from healthy donors reported mixed effects of FMT in alleviating PD symptoms. Ten of the patients received FMT via colonoscopy and 5 of the patients received FMT via nasointestinal route. Colonic route appeared superior, some of the patients reported improved health status for up to 24 months after FMT, although gut microbiota changes were not examined. However, no control or placebo group was included (28).

More than 70% of PD patients suffer from constipation affecting their quality of life. Recent study included 11 PD patients with constipation that underwent single FMT from healthy donors via nasoduodenal tube in order to alleviate gastrointestinal symptoms. Patients were evaluated 6 and 12 weeks after the first FMT. Stool was collected pre-FMT at 4, 6, 8, and 12 weeks after FMT, afterwards 16S rDNA sequencing for microbiome analysis was performed. Overall the gut microbiome diversity was lower in pre-FMT samples and increased post-FMT. In pre-FMT samples increased abundance of *Bacteroides* and reduced abundance of *Faecalibacterium* was observed. At 12 weeks post-FMT abundance of these genera reversed. Abundance of *Blautia*, a butyrate producing bacteria, increased post-FMT. Increased levels of butyrate could explain decreased gastrointestinal symptoms, however, this hypothesis needs to be proven. Baseline gut microbiome showed high relative abundance of *Enterobacteriaceae* which was positively correlated with postural instability and gait difficulty (79). After FMT the abundance of *Enterobacteriaceae* decreased with improvement in postural instability and gait difficulty. Similarly as in previous study, no control group was included (29).

## Conclusions

Advances in biotechnology and expansion of the knowledge on mechanisms of FMT have extended the spectrum of diseases treatable using FMT. Besides the well-known gastrointestinal indications such as *Clostridium difficile* infection, inflammatory bowel disease and irritable bowel syndrome that have been under massive clinical investigation for at least a decade, seemingly non-gastrointestinal disorders recently emerged as potential therapeutic targets for FMT. Dysbiosis was found in a number of metabolic, inflammatory, cardiovascular or neurological disorders, however, only a small number of clinical studies investigating the therapeutic effect of FMT have been published to date. Despite several methodological shortcomings, mostly positive results of these clinical studies indicate that further investigation of FMT as a therapeutic tool for non-gastrointestinal disorders can be expected in upcoming years.

## Author Contributions

RL and BG: conceptualization, writing, original draft preparation. RG: writing—review and editing, funding, and supervision. All authors have read and agreed to the published version of the manuscript.

## Funding

This research was funded by the Slovak Research and Development Agency under the contract no. APVV-17-0505 and by the Ministry of Education, Science, Research and Sport of the Slovak Republic under the contract no. VEGA 1/0649/21.

## Conflict of Interest

The authors declare that the research was conducted in the absence of any commercial or financial relationships that could be construed as a potential conflict of interest.

## Publisher's Note

All claims expressed in this article are solely those of the authors and do not necessarily represent those of their affiliated organizations, or those of the publisher, the editors and the reviewers. Any product that may be evaluated in this article, or claim that may be made by its manufacturer, is not guaranteed or endorsed by the publisher.
